# Supplementary material for: Chloroquine Enhances Death in Lung Adenocarcinoma A549 Cells Exposed to Cold Atmospheric Plasma Jet
Source: Cells. 2023 Jan 12;12(2):290. doi: 10.3390/cells12020290 (PMC9857254; doi:10.3390/cells12020290)
Supplement: Supplementary file 1 [file cells-12-00290-s001.zip › cells-2056297-supplementary.pdf]

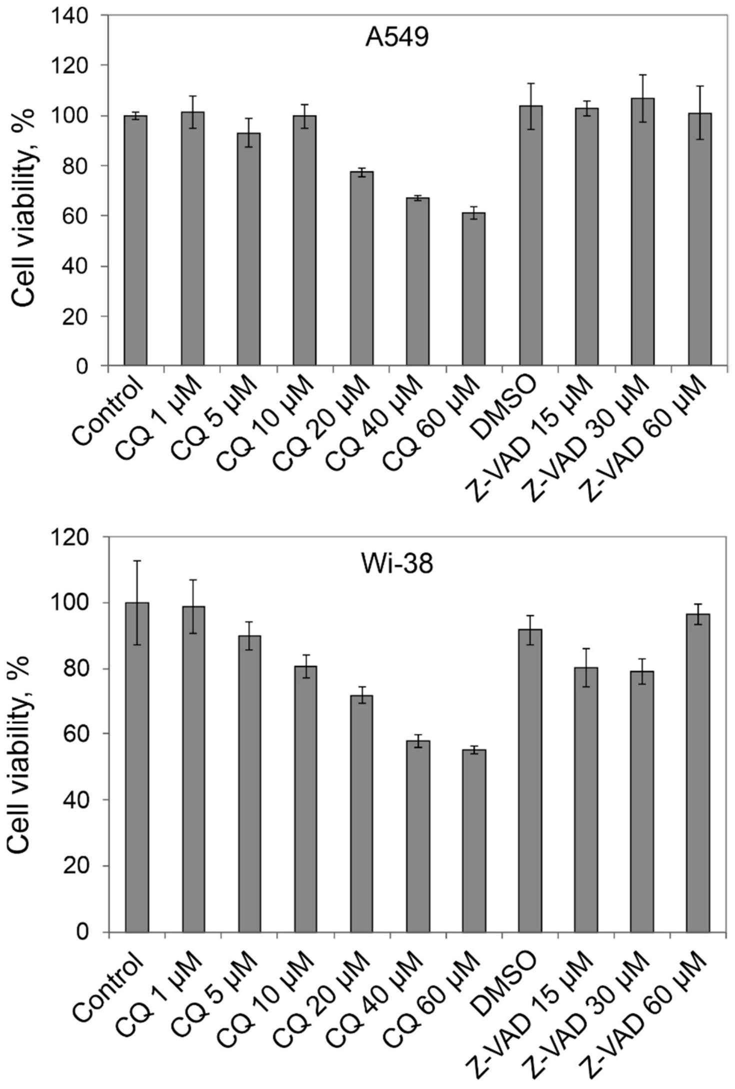

Figure S1. title: MTT analysis data of viability for Z-Vad and CQ-treated A549 and Wi-38 cells performed 24 h post-irradiation.

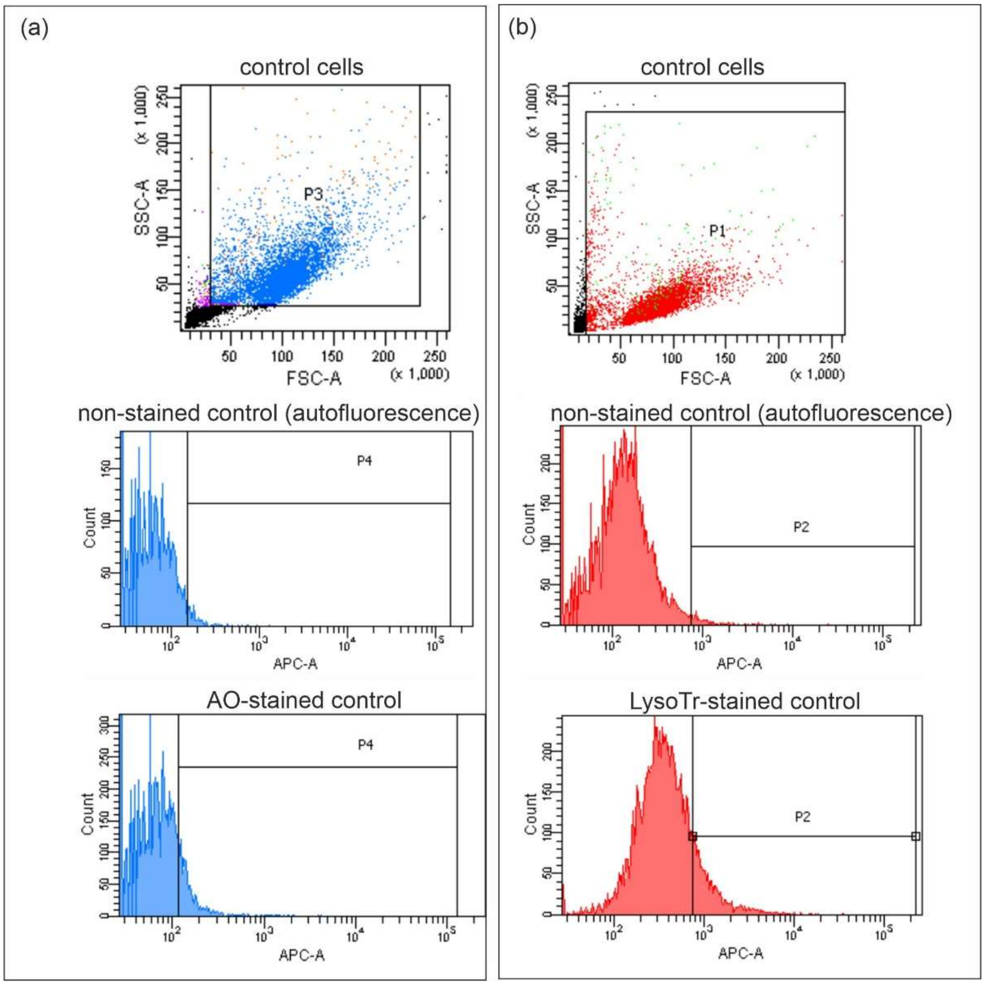

Figure S2. title: An example of gating in the forward and side-scatter channels and gating of the target population in A549 cells. (a) - analysis of AO-positive cells (P4); (b) - analysis of LysoTr-positive cells (P2). P3 and P1 populations were chosen so as to exclude cell debris from the analysis. P4 and P2 populations were chosen considering cells without added fluorochrome (non-stained control), so as to exclude autofluorescence.

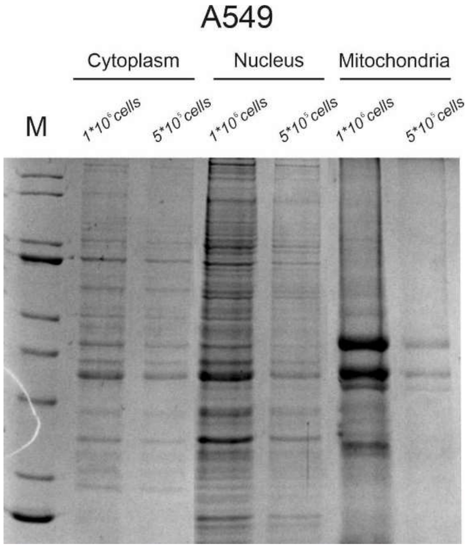

Figure S3. title: Analysis of the protein samples quality after fractionation.

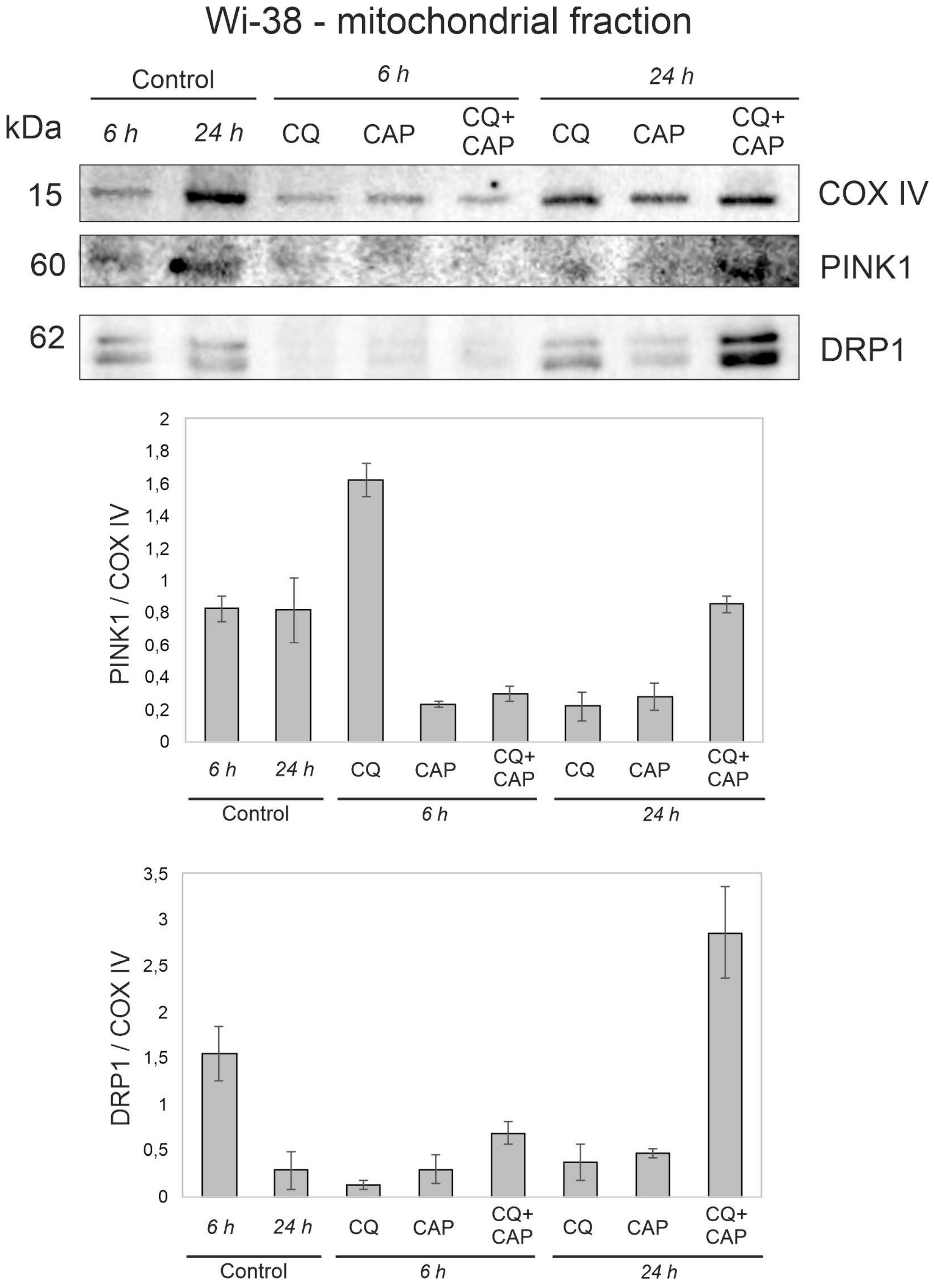

Figure S4. title: Changes in the mitochondrial proteins after CAP treatment in the Wi-38 cells. Representative western blots showing the changes in PINK1 and Drp1 proteins. Mitochondrial fraction was analyzed and COX IV was usedas a loading control. Wi-38 cells were exposed to CAP for 1 min (voltage 3.5 kV; voltage amplitude 50/4 kHz, helium flow 9 L/min). CQ was added alone to the cells or after CAP treatment to the final concentration 20 μM.
